# Effectiveness of the prevention of HIV mother -to-child transmission (PMTCT) program via early infant diagnosis (EID) data in Senegal

**DOI:** 10.1371/journal.pone.0215941

**Published:** 2019-05-21

**Authors:** Sokhna Bousso Gueye, Halimatou Diop-Ndiaye, Ousmane Diouf, Aissatou Sow-Ndoye, Fatoumata Touré, Ndèye Fatou Ngom-Faye, Diabou Diagne-Gueye, Khady Mbow-Ndiaye, Papa Amadou Niang Diallo, Aïssatou Gaye-Diallo, Souleymane Mboup, Cheikh Tidiane Ndour, Cheikh Saad-Bouh Boye, Coumba Touré-Kane

**Affiliations:** 1 Laboratoire Bactériologie-Virologie, CHU Aristide Le Dantec, Université Cheikh Anta Diop de Dakar, Dakar, Senegal; 2 Institut de Recherche en Santé, de Surveillance Epidémiologique et de Formations (IRESSEF), Diamniado, Senegal; 3 Division de la Lutte contre le Sida et les IST (DLSI), Ministère de la Santé et de l’Action Sociale, Dakar, Senegal; 4 United Nations Children’s Fund - UNICEF Senegal, Dakar, Senegal; 5 Conseil National de Lutte contre le Sida (CNLS), Primature, Dakar, Senegal; University of North Carolina at Chapel Hill, UNITED STATES

## Abstract

**Background:**

To improve the care and treatment of HIV-exposed children, early infant diagnosis (EID) using dried blood spot (DBS) sampling has been performed in Senegal since 2007, making molecular diagnosis accessible for patients living in decentralized settings. This study aimed to determine the evolution of the HIV transmission rate in children from 2008 to 2015 and to analyze associated factors, particularly the mother’s treatment status and/or child’s prophylaxis status and the feeding mode.

**Methods:**

The data were analyzed using EID reports from the reference laboratory. Information related to sociodemographic characteristics, HIV profiles, the mother’s treatment status, the child’s prophylaxis status, and the feeding mode was included. Descriptive statistics were calculated, and bivariate and multivariate logistic regression analyses were performed.

**Results:**

During the study period, a total of 5418 samples (5020 DBS and 398 buffy coat) from 168 primary prevention of HIV mother-to-child transmission (PMTCT) intervention sites in Senegal were tested. The samples were collected from 4443 children with a median age of 8 weeks (1–140 weeks) and a sex ratio (M/F) of 1.1 (2309/2095). One-third (35.2%; N = 1564) of the children were tested before 6 weeks of age. Twenty percent (N = 885) underwent molecular diagnostic testing more than once. An increased number of mothers receiving treatment (57.4%; N = 2550) and children receiving prophylaxis (52.1%; N = 2315) for protection against HIV infection during breastfeeding was found over the study period. The transmission rate decreased from 14.8% (95% confidence interval (CI): 11.4–18.3) in 2008 to 4.1% (95% CI: 2.5–7.5) in 2015 (p < 0.001). However, multivariate logistic regression analysis revealed that independent predictors of HIV mother-to-child transmission included lack of mother’s treatment (adjusted odd ratio (aOR) = 3.8, 95% CI: 1.9–7.7; p˂0.001), lack of child’s prophylaxis (aOR = 7.8, 95% CI: 1.7–35.7; p = 0.009), infant age at diagnosis (aOR = 2.2, 95% CI: 1.1–4.3 for ≤6 weeks versus 12–24 weeks; p = 0.025) and protective effect of breastfeeding on ART against formula feeding (aOR = 0.4, 95% CI: 0.2, 0.7; p = 0.005).

**Conclusion:**

This study demonstrates the effectiveness of PMTCT interventions in Senegal but indicates also that increased efforts should be continued to reduce the MTCT rate to less than 2%.

## Introduction

Pediatric HIV infection remains a significant public health issue; 2.6 million children, 2.3 million of whom were in sub-Saharan Africa (SSA), were infected worldwide in 2014 [1]. To reduce HIV mother-to-child transmission (MTCT), different strategies have been recommended by the World Health Organization (WHO).

**Fig 1:**
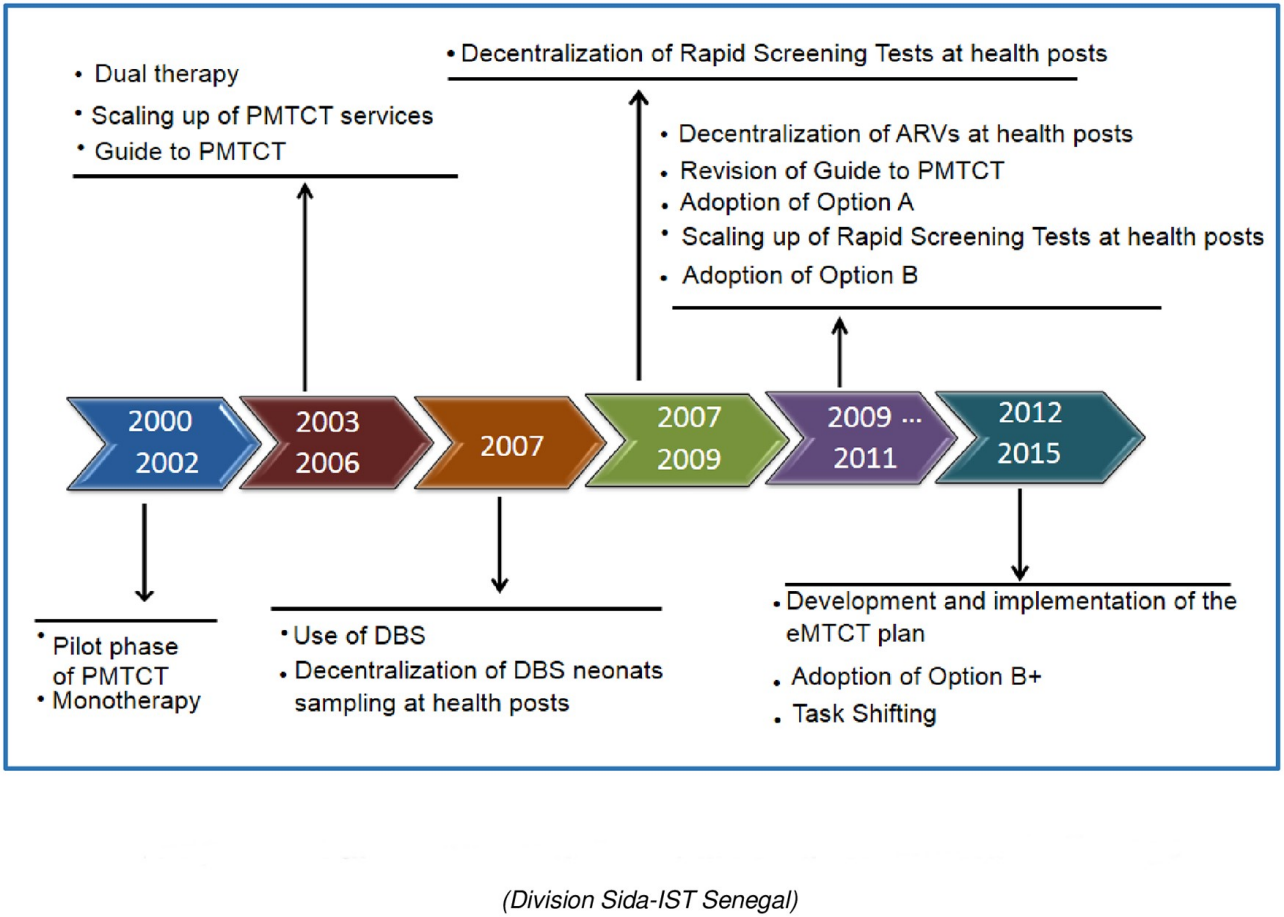
Expansion and improvement in PMTCT interventions in Senegal. (Division Sida-IST Senegal).

Adoption of WHO Options (Fig 1) was from the initial regimen of monotherapy with zidovudine (AZT) at 36 weeks of pregnancy and highly active antiretroviral therapy (HAART) at 14 weeks before delivery (Option A) to the more recent Option B and Option B+. In alignment with the WHO guidelines, Option A was adopted in Senegal in 2010; in this regimen, AZT treatment is begun at the 14^th^ week of gestation, a single dose of nevirapine (sdNVP) is provided during labor, daily doses of zidovudine/lamivudine (AZT/3TC) are given for 7 days postpartum, and finally, daily doses of NVP are given from birth to up to 4–6 weeks postpartum. By the end of 2011, Option B was adopted; this option consists of a three-drug regimen provided to the mother from the 14^th^ week of gestation to delivery and continuing during the entire breastfeeding period. Furthermore, prophylactic treatment is provided to newborn infants as part of Option B. At the end of 2012, Senegal adopted Option B+, which provides lifelong ART to all HIV-infected pregnant and breastfeeding women irrespective of CD4 count or clinical stage [2–4]. To prevent HIV infection and ensure the survival of infants, ART during the breastfeeding period is recommended until the infant is 12 months of age, based on evolving WHO guidelines.

Other WHO strategies (Fig 1) implemented in the country to reduce HIV MTCT include scaling up prevention of MTCT (PMTCT) services, providing rapid screening for pregnant women, and using dried blood spot (DBS) sampling for early infant diagnosis (EID) in decentralized settings (Fig 1). EID should be routinely performed in all children aged 4 to 6 weeks born to HIV-infected mothers [5, 6] to allow and inform the initiation of appropriate lifesaving treatment. The early provision of ART has been shown to have the potential to save the lives of ≥ 50% of children infected by HIV by the time they reach 2 years of age [7–9].

In Senegal, PMTCT sites and use of DBS increased (Fig 1) according to WHO guidelines. The use of DBS sampling for EID became policy in 2007 and scaled rapidly across the country, thus becoming more widely accessible. In 2006, only 61 sites throughout the country used DBS sampling for EID. However, in 2014, DBS sampling was performed at 1045 PMTCT sites, which were classified as primary sites (hospitals and sanitary districts) and secondary sites (health posts). [10, 11].

These improvements were in line with the WHO recommendations and goal of achieving an MTCT rate lower than 5% by 2015 considered as a virtual elimination of MTCT (virtual eMTCT).

The objective of this study was to evaluate the Senegalese PMTCT response from 2008 to 2015 using EID data and to measure the impact of related efforts on decreasing the HIV MTCT rate.

## Material and methods

### Data collection

Data from EID request forms and EID report results collected at the bacteriology-virology reference laboratory of Le Dantec hospital in Dakar from 2008 to 2015 were analyzed. These data were programmatic efforts and included information routinely collected in mother/child medical records, such as age, site visits, mother’s HIV status and treatment status, child’s prophylaxis status, feeding mode, and type of samples collected (both whole blood in EDTA tubes and DBS samples).

**Fig 2:**
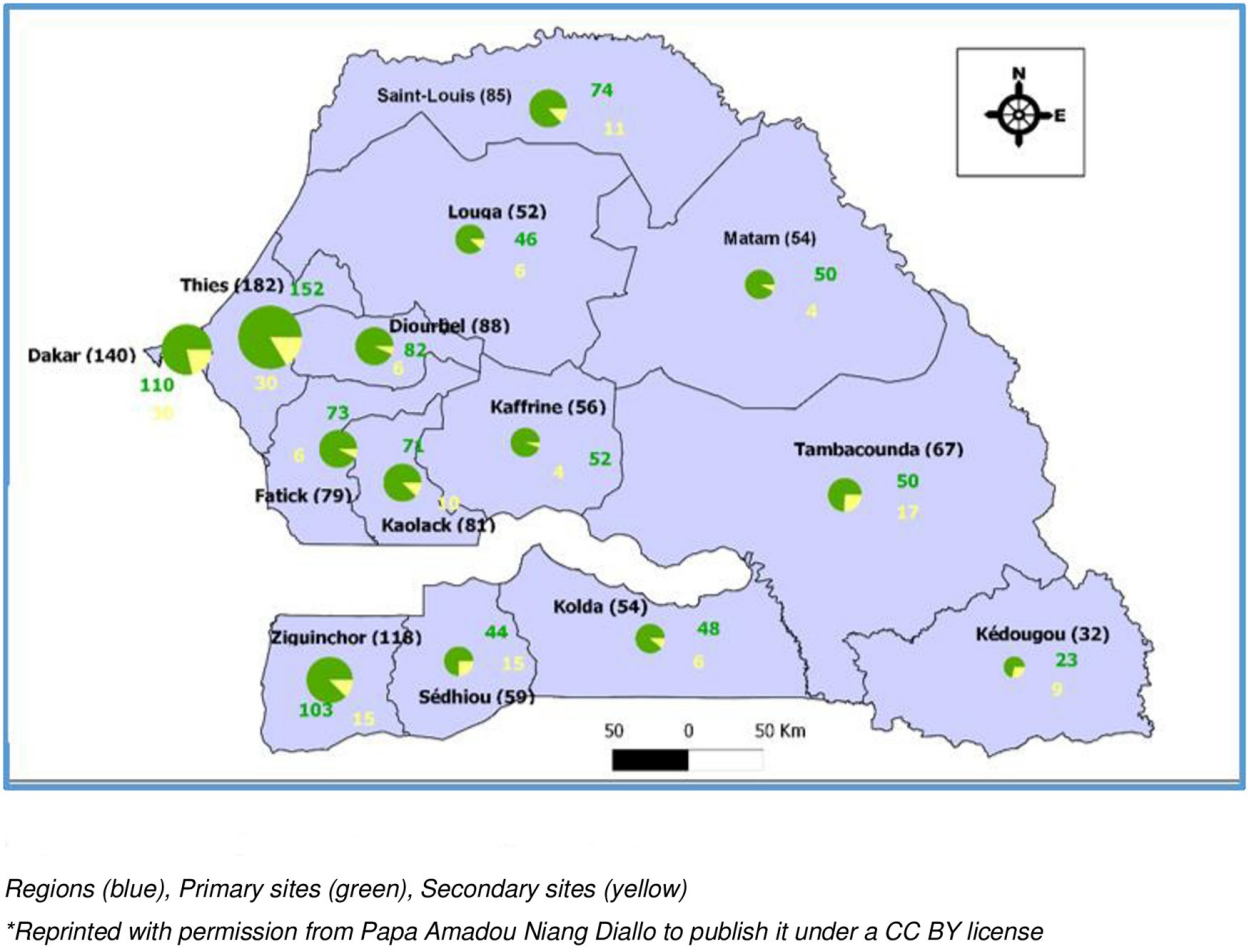
Senegalese PMTCT sites in 2015. * Cheikh Anta Diop University (CADU)/Afriqualab PT Program: is an external quality assurance/Proficiency Testing for HIV Rapid Tests, EID & Viral Load, technologies transferred by Centers for Disease Control and Prevention (CDC), Atlanta, USA.

**Fig 3:**
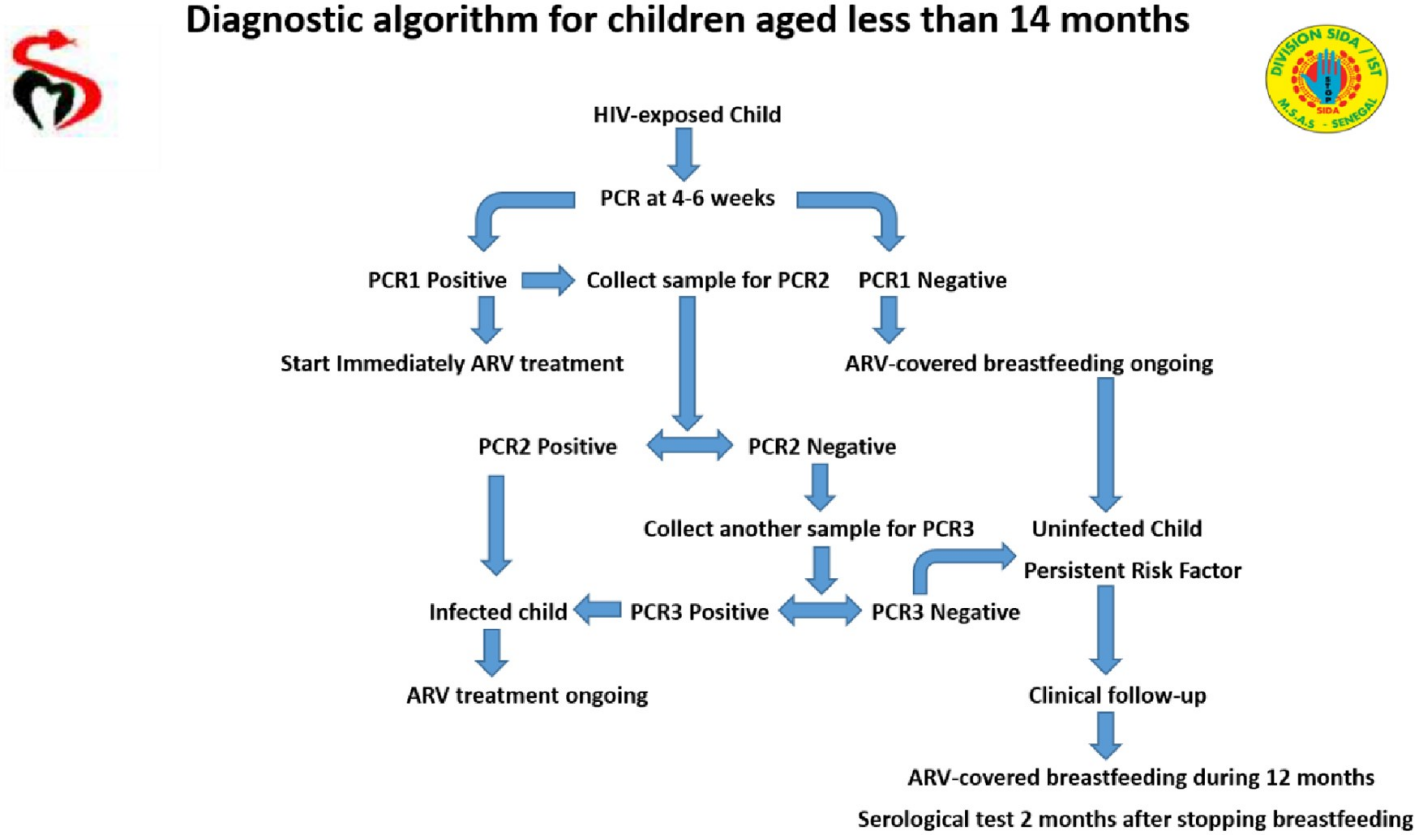
Senegalese algorithm of early infant diagnosis. Regions (blue), Primary sites (green), Secondary sites (yellow), Reprinted with permission from Papa Amadou Niang Diallo to publish it under a CC BY license.

Blood samples from children born to HIV-seropositive mothers were collected from different PMTCT sites throughout the country. The number of sites reported increased from 258 in 2008 to 1146 in 2015, including 168 primary and 978 secondary sites (Fig 2). Whole blood was collected from Dakar pediatric reference centers, and DBS samples were collected from some Dakar PMTCT sites and from other regions of Senegal. After collection, samples were sent with the standard EID request form to the reference laboratory, an ISO 15189 accredited medical laboratory, where PCR tests, under CDC-CADU/Afriqualab PT Program*, were performed to determine the infant’s HIV status. All positive results were confirmed with a second test using another sample according to the EID algorithm before being entered in the local electronic laboratory database (Fig 3). This database was used to calculate the annual HIV prevalence and to analyze factors associated with HIV infection, such as the mother’s treatment status and/or the child’s prophylaxis status and the feeding mode. Four feeding modes were reported: formula feeding; mixed feeding, which combines breast and formula feeding; exclusive breastfeeding; and breastfeeding on ART, which consists of breastfeeding for 12 months postpartum to lower the MTCT risk while the mother is on ART.

### Statistical analysis

The data were entered into a Microsoft Excel database and analyzed using SPSS version 16 and STATA version 12. Descriptive statistics were calculated for selected characteristics and the MTCT rates were estimated in proportion with 95% confidence intervals (CI), using Wald method [12]. Bivariate and multivariate logistic regression analyses to determine associations between HIV testing results and other variables were performed. Odd ratio (OR) with a 95% CI was used to measure the degree of association with infant HIV positivity. Bivariate analysis was performed with univariate logistic regression or cross tabulation analysis and multivariate analysis was used to calculate the adjusted odd ratio and addressed the confounding issues. The Chi-square test was used to estimate the difference between proportions in successive years. For Chi-square values lower than 0.05, Fisher’s exact test was also used to estimate the *p*-value. *p*-values less than 0.05 were considered statistically significant.

## Results

### Sample distribution by region

This MTCT analysis was carried out on 5418 samples comprising 398 venous blood samples from Dakar pediatric reference centers and 5020 DBS samples from Dakar and from PMTCT sites in other regions. The distance from the sites to the reference laboratory ranged from 14 to 682 km (Fig 2). The samples were mainly from Dakar and Ziguinchor; 43.2% (2338) and 13% (704) of the samples, respectively, were from these cities (Table 1).

**Table 1:** Distribution of PMTCT sites and the numbers of samples and children tested by region.

Region		Number of	Samples	Children	Infected children
		PMTCT sites	N (%)	N (%)	N (%*)
Dakar	PRC*	3	398 (7.4)	325 (7.3)	50 (15.4)
	OPS*	137	1940 (35.8)	1540 (34.7)	90 (5.8)
Diourbel		182	241 (4.5)	212 (4.8)	9 (4.2)
Fatick		88	137 (2.5)	114 (2.6)	7 (6.1)
Kaffrine		52	60 (1.1)	55 (1.2)	2 (3.6)
Kaolack		85	297 (5.5)	272 (6.1)	19 (7.0)
Kédougou		54	59 (1.1)	55 (1.2)	2 (3.6)
Kolda		79	178 (3.3)	158 (3.6)	11 (7.0)
Louga		81	180 (3.3)	142 (3.2)	9 (6.3)
Matam		55	54 (1.0)	53 (1.2)	5 (9.4)
Saint louis		67	346 (6.4)	291 (6.5)	18 (6.2)
Sédhiou		32	228 (4.2)	201 (4.5)	10 (5.0)
Tambacounda		54	230 (4.2)	194 (4.4)	7 (3.6)
Thiès		59	349 (6.4)	279 (6.3)	26 (9.3)
Ziguinchor		118	704 (13.0)	538 (12.1)	27 (5.0)
UNK		0	17 (0.3)	14 (0.3)	0
**TOTAL**		**1146**	**5418**	**4443**	**292**

Dakar PRC*: pediatric reference centers (receiving children sick enough to warrant testing); Dakar OPS*: other PMTCT sites; UNK*: unknown; %*: percentage of infected children reported, with the total number of children tested as the denominator

### Characteristics of the children

Samples were collected from 4443 children, with more than one sample tested from 885 of these children. The median age was 8 weeks and ranged from 1 week to 24 months; the sex ratio (M/F) was 1.1 (2309/2095). The characteristics of the children and mothers over the study period are presented in Table 2. The greatest increase in mothers receiving treatment and children receiving prophylaxis to protect against MTCT through breastfeeding occurred between 2010 and 2012.

**Table 2:** Characteristics of the mother-child pairs in the PMTCT interventions.

Characteristics	2008	2009	2010	2011	2012	2013	2014	2015	Total	p-value
	n (%)	n (%)	n (%)	n (%)	n (%)	n (%)	n (%)	n (%)	n (%)	
**Sex**	**N = 411**	**N = 494**	**N = 554**	**N = 555**	**N = 630**	**N = 594**	**N = 623**	**N = 582**	**N = 4443**	
Female	171 (41.6)	236 (47.8)	261 (47.1)	262 (47.2)	294 (46.7)	296 (49.8)	296 (47.5)	279 (47.9)	2095 (47.1)	
Male	222 (54.0)	253 (51.2)	284 (51.3)	291 (52.4)	336 (53.3)	293 (49.3)	327 (52.5)	303 (52.1)	2309 (52.0)	
UNK	18 (4.4)	5 (1.0)	9 (1.6)	2 (0.4)	0	5 (0.8)	0	0	39 (0.9)	
**Age at first PCR in weeks**	**N = 411**	**N = 494**	**N = 554**	**N = 555**	**N = 630**	**N = 594**	**N = 623**	**N = 582**	**N = 4443**	
≤ 6	47 (11.4)	131(26.5)	179 (32.3)	230 (41.4)	271 (43.0)	266 (44.8)	239 (38.3)	201 (34.6)	1564 (35.2)	<0,001
6–12	49 (11.9)	132 (26.7)	131 (23.7)	116 (20.9)	134 (21.3)	133 (22.4)	181 (29.1)	183 (31.5)	1059 (23.8)	<0,001
12–24	42 (10.2)	89 (18.1)	107 (19.3)	82 (14.8)	98 (15.6)	96 (16.2)	103 (16.5)	98 (16.8)	715 (16.1)	0,322
24–48	29 (7.1)	61 (12.4)	81 (14.6)	96 (17.3)	91 (14.5)	67 (11.3)	31 (5.0)	67 (11.5)	523 (11.8)	0,194
48–72	20 (4.9)	18 (3.6)	20 (3.6)	21 (3.8)	16 (2.5)	21 (3.5)	31 (5.0)	9 (1.5)	156 (3.5)	0.085
˃ 72	4 (1.0)	4 (0.8)	8 (1.4)	2 (0.4)	4 (0.6)	0	20 (3.2)	2 (0.3)	44 (1.0)	0,054
UNK	220 (53.5)	59 (11.9)	28 (5.1)	8 (1.4)	16 (2.5)	11 (1.8)	18 (2.9)	22 (3.8)	382 (8.6)	0,022
**Mother’s treatment status**	**N = 411**	**N = 494**	**N = 554**	**N = 555**	**N = 630**	**N = 594**	**N = 623**	**N = 582**	**N = 4443**	
Yes	-	1 (0.2)	83 (15.0)	389 (70.1)	508 (80.6)	512 (86.2)	541 (86.8)	516 (88.7)	2550 (57.4)	<0.001
No	-	-	7 (1.2)	41 (7.4)	54 (8.6)	52 (8.8)	54 (8.7)	36 (6.2)	244 (5.5)	0.675
UNK	411 (100)	493 (99.8)	464 (83.8)	125 (22.5)	68 (10.8)	30 (5.0)	28 (4.5)	30 (5.1)	1649 (37.1)	0,021
**Mother’s treatment regimen**	**-**	**N = 1**	**N = 83**	**N = 389**	**N = 508**	**N = 512**	**N = 541**	**N = 516**	**N = 2550**	
Monotherapy	-	1(100)	2 (2.4)	3 (0.8)	-	-	-	-	5 (0.2)	0.654
Option A	-	-	72 (86.7)	326 (83.8)	5 (1.0)	-	-	-	404 (15.9)	<0.001
Option B	-	-	-	30 (7.7)	402 (79.1)	69 (13.5)	4 (0.8)	-	505 (19.8)	<0.001
Option B+	-	-	-	-	59 (11.6)	358 (69.9)	486 (89.8)	467 (90.5)	1370 (53.7)	<0.001
UNK	-	-	9 (10.9)	30 (7.7)	42 (8.3)	85 (16.6)	51 (9.4)	49 (9.5)	266 (10.4)	0.003
**Child’s prophylaxis status**	**N = 411**	**N = 494**	**N = 554**	**N = 555**	**N = 630**	**N = 594**	**N = 623**	**N = 582**	**N = 4443**	
Yes	-	1 (0.2)	84 (15.2)	386 (69.5)	448 (71.1)	450 (75.7)	484 (77.7)	462 (79.4)	2315 (52.1)	<0.001
No	-	-	11 (2)	72 (13.0)	115 (18.3)	80 (13.5)	99 (15.9)	90 (15.5)	467 (10.5)	0.246
UNK	411 (100)	493 (99.8)	459 (82.8)	97 (17.5)	67 (10.6)	64 (10.8)	40 (6.4)	30 (5.1)	1661 (37.4)	0.028
**Child’s prophylaxis regimen**	**-**	**N = 1**	**N = 84**	**N = 386**	**N = 448**	**N = 450**	**N = 484**	**N = 462**	**N = 2315**	
Monotherapy	-	1 (100)	3 (3.6)	5 (1.3)	-	-	-	-	9 (0.4)	0.465
Option A	-	-	67 (79.7)	319 (82.6)	11 (2.5)	-	-	-	397 (17.1)	<0.001
Option B	-	-	-	35 (9.1)	340 (75.9)	52 (11.6)	3 (0.6)	-	430 (18.6)	<0.001
Option B+	-	-	-	-	71 (15.8)	365 (81.1)	460 (95.1)	445 (96.3)	1341 (57.9)	<0.001
UNK	-	-	14 (16.7)	27 (7.0)	26 (5.8)	33 (7.3)	21 (4.3)	17 (3.7)	138 (6.0)	0.022
**Feeding mode**	**N = 411**	**N = 494**	**N = 554**	**N = 555**	**N = 630**	**N = 594**	**N = 623**	**N = 582**	**N = 4443**	
Formula feeding	-	-	1 (0.2)	17 (3.0)	40 (6.3)	36 (6.1)	38 (6.1)	44 (7.6)	176 (4.0)	0.006
Mixed feeding	-	-	-	7 (1.3)	21 (3.3)	25 (4.2)	24 (3.8)	9 (1.5)	86 (1.9)	0.120
Exclusive breastfeeding	-	-	-	26 (4.7)	122 (19.4)	98 (16.5)	74 (11.9)	54 (9.3)	374 (8.4)	0.098
Breastfeeding on ART	-	-	2 (0.4)	151 (27.2)	393 (62.4)	378 (63.6)	451 (72.4)	454 (78.0)	1829 (41.2)	<0.001
UNK	411 (100)	494 (100)	551 (99.4)	354 (63.8)	54 (8.6)	57 (9.6)	36 (5.8)	21 (3.6)	1978 (44.5)	0.011

UNK*: unknown; N*: denominator; n*: numerator

### Prevalence of HIV

**Fig 4:**
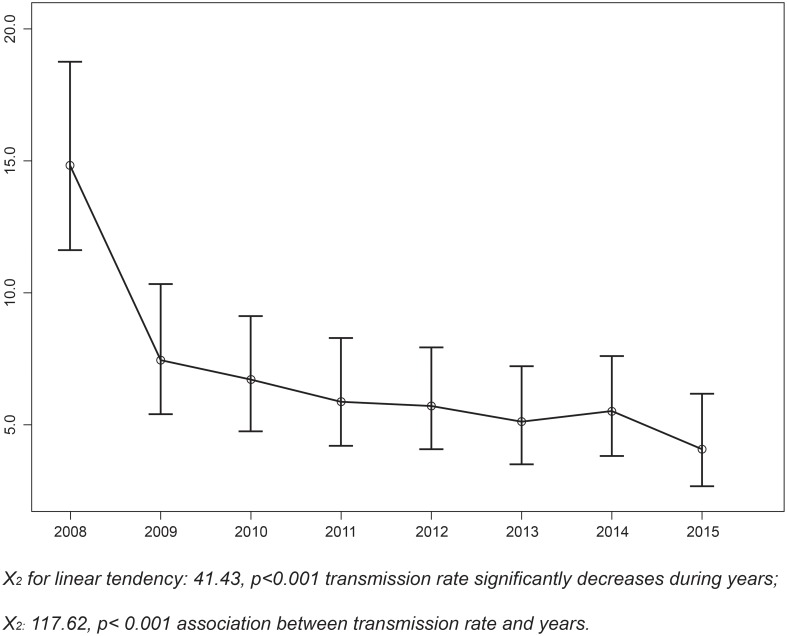
MTCT rate (%) between 2008 and 2015. X_2_ for linear tendency: 41.43, p<0.001 transmission rate significantly decreases during years; X_2:_ 117.62, p< 0.001 association between transmission rate and years.

The number of samples from children receiving EID testing increased from 411 (70.6%) in 2008 to 494 (72%) in 2009. The HIV transmission rate between these two years decreased from 14.8% (95% CI: 11.4–18.3) to 7.5% (95% CI: 5.2–9.8). The MTCT rate continued to decrease through 2015, when it dropped below 5% for the first time in Senegal (Fig 4; Table 3).

**Table 3:** Number of PMTCT sites, number and percentage of samples, number of tested children, and MTCT rate by year.

Year	Number of PMTCT sites	Number and percentage of samples N (%)	Number of tested children	Transmission rate	95% CI
**2008**	258	582 (70,6)	411	61 (14.8%)	(11.4–18.3)
**2009**	503	686 (72,0)	494	37 (7.5%)	(5.2–9.8)
**2010**	648	788 (70,3)	554	37 (6.7%)	(4.6–8.7)
**2011**	707	691 (80,3)	555	33 (5.9%)	(3.9–7.9)
**2012**	1009	720 (87,5)	630	36 (5.7%)	(3.9–7.5)
**2013**	1027	665 (89,3)	594	30 (5.1%)	(3.3–6.8)
**2014**	1045	677 (92,0)	623	34 (5.5%)	(3.7–7.2)
**2015**	1146	609 (95,6)	582	24 (4.1%)	(2.5–5.7)

The number of children being tested increased in all age categories below 48 weeks by 2009, and the proportion of tested children under 12 weeks of age climbed to 60–65% by 2015 (Table 2). Overall, the first diagnostic test was performed before 12 weeks of age in 59% of the children (2623/4443). It was found that the proportion of children tested before 12 weeks of age increased significantly (p-values˂0.05) over the study period while it decreased among those tested after 12 weeks of age (p-values˃0.05) (Table 2). A coincident decrease in MTCT (˂ 5%) occurred between 2008 and 2009 in children tested between 6 and 24 weeks of age, and a slower decrease in MTCT (˂ 3%) occurred among children tested before 6 weeks of age (Table 4). Children tested after 12 weeks of age presented higher transmission rates than did those tested before 12 weeks of age in the same period (Table 4).

**Table 4:** MTCT rate according to age, feeding mode, treatment, and prophylaxis regimens.

Characteristics	2008	2009	2010	2011	2012	2013	2014	2015	Total	p-value (trend: 2008–2015)
	n (%) N	n (%) N	n (%) N	n (%) N	n (%) N	n (%) N	n (%) N	n (%) N	n (%) N	
**Sex**										
Male	31 (14.0) 222	14 (5.5) 253	19 (6.7) 283	22 (7.6) 291	15 (4.5) 336	20 (6.8) 293	19 (5.8) 327	16 (5.3) 303	156 (3.5) 2308	0.004
Female	25 (14.6) 171	23 (9.7) 236	17 (6.5) 260	11 (4.2) 262	21 (7.1) 294	10 (3.4) 295	15 (5.1) 296	8 (2.9) 279	130 (2.9) 2093	<0.001
**Age in weeks**										
≤ 6	2 (4.3) 47	7 (5.3) 131	4 (2.2) 179	3 (1.3) 230	13 (4.8) 271	3 (1.1) 266	2 (0.8) 239	3 (1.5) 201	37 (2.4) 1564	0.016
6–12	9 (18.4) 49	5 (3.8) 132	4 (3.1) 131	5 (4.3) 116	3 (2.2) 134	5 (3.8) 133	8 (4.4) 181	6 (3.3) 183	45 (4.2) 1059	0.051
12–24	13 (30.9) 42	4 (4.5) 89	10 (9.3) 107	5 (6.1) 82	6 (6.1) 98	9 (9.4) 96	9 (8.7) 103	5 (5.1) 98	61 (8.5) 715	0.028
24–48	6 (20.7) 29	11 (18.0) 61	9 (11.1) 81	11 (11.5) 96	12 (13.2) 91	10 (14.9) 67	3 (9.7) 31	5 (7.5) 67	67 (12.8) 523	0.093
48–72	4 (20.0) 20	3 (16.7) 18	4 (21.1) 19	8 (38.1) 21	0 (0.0) 16	2 (18.2) 11	7 (22.6) 31	3 (33.3) 9	31 (21.4) 145	0.728
> 72	2 (50.0) 4	1 (25.0) 4	0 (0.0) 8	0 (0.0) 1	0 (0.0) 4		2 (10.0) 20	0 (0.0) 2	5 (11.6) 43	0.099
**Feeding mode**										
Formula feeding			0 (0.0) 1	2 (11.8) 17	10 (25.0) 40	0 (0.0) 36	5 (13.2) 38	6 (13.6) 44	23 (13.1) 176	0.518
Mixed feeding				1 (14.3) 7	2 (9.5) 21	8 (32.0) 25	4 (16.7) 24	2 (22.2) 9	17 (19.8) 86	0.531
Exclusive breastfeeding				0 (0.0) 26	9 (7.4) 122	6 (6.1) 98	11 (14.9) 74	4 (7.4) 54	30 (8.0) 374	0.391
Breastfeeding on ART			0 (0.0) 2	4 (2.6) 151	10 (2.5) 393	10 (2.6) 378	12 (2.7) 451	10 (2.2) 454	46 (2.5) 1829	0.752
**Mother’s** **treatment status**										
Yes		0 (0.0) 1	1 (1.1) 93	17 (4.2) 409	13 (2.5) 521	13 (2.7) 485	16 (3.0) 541	14 (2.7) 516	74 (2.9) 2566	0.707
No			2 (28.6) 7	5 (12.2) 41	17 (31.5) 54	12 (23.1) 52	15 (27.8) 54	8 (22.2) 36	59 (24.2) 244	0.557
**Child’s** **prophylaxis status**										
Yes		0 (0.0) 1	1 (1.2) 84	10 (2.6) 386	10 (2.2) 448	9 (2.0) 449	13 (2.7) 484	6 (1.3) 462	49 (2.1) 2314	0.517
No			3 (42.9) 7	10 (15.2) 66	21 (18.4) 114	13 (16.2) 80	18 (18.2) 99	15 (16.7) 90	80 (17.1) 456	0.044
**Mother’s** **treatment-child’s prophylaxis status**										
Yes		0 (0.0) 1	1 (1.4) 73	9 (2.7) 328	8 (1.9) 432	8 (1.9) 430	13 (2.8) 470	6 (1.4) 441	45 (2.1) 2175	0.611
No			3 (8.6) 35	14 (8.5) 165	24 (16.0) 150	17 (14.5) 117	19 (14.3) 133	18 (13.7) 131	95 (13.0) 731	0.182

% number of HIV-positive children **(n)** divided by the total number of children tested **(N)** in the indicated age range in the indicated year

In addition, Table 2 shows an improvement in prophylaxis over the study period, with an increase in the number of mothers treated and/or children given prophylaxis of approximately 70–88% since 2011. In addition, the proportion of infected children in each group is 6 to 10 times lower when the mother was treated and/or the child was given prophylaxis than the absence of treatment and/or prophylaxis ie (the mother was not treated and/or the child was not given prophylaxis) (Table 4).

According to the analysis of the infant feeding mode, breastfeeding on ART increased over the study period, from 60% in 2012 to 78% in 2015 (Table 2). In addition, beginning in 2012, less than 3% of infants breastfed on ART were infected (Table 4).

The factors associated with HIV infection in children in the bivariate analysis were a late age at testing, feeding with the exclusive/mixed modes, and the lack of the mother’s treatment/child’s prophylaxis; the risk associated with these factors decreased over the study period, especially in 2009/2012, 2012/2015, and 2011, respectively (Table 5).

**Table 5:** Factors associated with HIV infection in the bivariate logistic regression analysis.

	2008	2009	2010	2011	2012	2013	2014	2015	Total	
Characteristics	OR (95% CI)	OR (95% CI)	OR (95% CI)	OR (95% CI)	OR (95% CI)	OR (95% CI)	OR (95% CI)	OR (95% CI)	OR (95% CI)	p-value
**Number of individual characteristics**	**411**	**494**	**554**	**555**	**630**	**594**	**623**	**582**	**4443**	
**Sex**										
Male	1.0	1.0	1.0	1.0	1.0	1.0	1.0	1.0	1.0	
Female	1.1 (0.6–1.9)	1.8 (0.9–3.7)	0.9 (0.5–1.9)	0.5 (0.2–1.1)	1.6 (0.8–3.3)	0.07 (0.05–0.1)	0.9 (0.4–1.7)	0.5 (0.2–1.3)	0.9 (0.5–1.9)	0.370
**Age in weeks**										
≤ 6	1.0	1.0	1.0	1.0	1.0	1.0	1.0	1.0	1.0	
6–12	5.1 (1.0–24.8)	0.7 (0.2–2.3)	1.4 (0.3–5.6)	3.4 (0.8–14.5)	0.5 (0.1–1.6)	3.4 (0.8–14.6)	5.5 (1.1–26.1)	2.2 (0.5–9.1)	2.8 (0.6–12.3)	0.245
12–24	10.1 (2.1–48.0)	0.8 (0.2–2.9)	4.5 (1.4–14.8)	4.9 (1.1–21.0)	1.3 (0.5–3.5)	9.1 (2.4–34.3)	11.3 (2.4–53.5)	3.5 (0.8–15.2)	5.7 (1.4–24.2)	0.001
24–48	5.9 (1.1–31.4)	3.9 (1.4–10.6)	5.5 (1.6–18.3)	9.8 (2.6–35.9)	3.0 (1.3–6.9)	15.4 (4.1–57.7)	12.7 (2.0–79.3)	5.3 (1.2–22.9)	7.7 (1.9–32.9)	0.012
48–72	5.6 (0.9–33.7)	3.5 (0.8–15.2)	11.6 (2.6–51.4)	46.6 (11.0–196.5)		19.5 (2.9–131.4)	34.6 (6.8–175.8)	33 (5.5–198.6)	22.1 (4.4–114.7)	0.019
> 72	22.5 (2.0–252.6)	5.9 (0.5–64.3)					13.2 (1.8–99.0)		13.9 (1.4–138.6)	0.021
**Feeding mode**										
Formula feeding				1.0	1.0		1.0	1.0	1.0	
Mixed feeding				1.25 (0.1–16.5)	0.3 (0.1–1.6)	17.3 (6.1–49.4)	1.3 (0.3–5.5)	1.8 (0.3–10.8)	4.4 (1.4–16.8)	<0.001
Exclusive breastfeeding					0.2 (0.1–0.6)	2.4 (0.9–6.7)	1.2 (0.4–3.6)	0.5 (0.1–1.9)	1.1 (0.4–3.2)	<0.001
Breastfeeding on ART				0.2 (0.03–1.2)	0.08 (0.03–0.2)		0.2 (0.06–0.5)	0.1 (0.05–0.4)	0.2 (0.04–0.6)	0.084
**Mother’s treatment status**										
Yes			1.0	1.0	1.0	1.0	1.0	1.0	1.0	
No			36.8 (2.8–477.7)	3.2 (1.1–9.2)	17.9 (8.1–39.7)	10.9 (4.6–25.5)	12.6 (5.8–27.4)	10.2 (3.9–26.4)	15.3 (4.4–101.0)	<0.001
**Child’s prophylaxis status**										
Yes			1.0	1.0	1.0	1.0	1.0	1.0	1.0	
No			62.2 (5.2–740.1)	6.7 (2.7–16.8)	9.9 (4.5–21.7)	9.5 (3.9–23.1)	8.0 (3.8–17.1)	15.2 (5.7–40.4)	18.6 (4.3–143.2)	<0.001
**Mother’s treatment-child’s prophylaxis status**										
Yes			1.0	1.0	1.0	1.0	1.0	1.0	1.0	
No			6.75 (0.7–67.4)	3.3 (1.4–7.8)	10.1 (4.4–23.0)	8.9 (3.8–21.4)	5.9 (2.8–12.2)	11.5 (4.5–29.8)	7.7 (2.9–26.9)	<0.001

Table 5 (bivariate analysis) and Table 6 (multivariate analysis) show that the sooner the better for the child’s HIV diagnosis, the better if the mother is under treatment, better if the child is on prophylaxis, better if breastfeeding is on antiretroviral treatment (ART). These factors showed to help reduce the MTCT rate.

**Table 6:** Multivariate logistic regression analysis of the association between HIV positivity and selected characteristics (5418 samples, 4443 children, 292 HIV-infected children).

Characteristics (N = 2253)	coefficient	Standard error	Adjusted OR (95% CI)	p-value
**Sex**				
Male	-	-	1.0	-
Female	-0.05	0.226	0.9 (0.6–1.5)	0.831
**Age in weeks**				
≤ 6	-	-	1.0	-
6–12	0.434	0.344	1.5 (0.8–3.0)	0.207
12–24	0.782	0.348	2.2 (1.1–4.3)	0.025
24–48	1.120	0.350	3.0 (1.5–6.0)	0.001
48–72	1.642	0.499	5.2 (1.9–13.8)	0.001
> 72	0.700	0.862	2.0 (0.4–10.9)	0.417
**Feeding mode**				
Formula feeding	-	-	1.0	-
Mixed feeding	-0.694	0.449	0.5 (0.2–1.2)	0.122
Exclusive breastfeeding	-1.189	0.375	0.3 (0.1–0.6)	0.002
Breastfeeding on ART	-1.011	0.357	0.4 (0.2–0.7)	0.005
**Mother’s treatment status**				
Yes	-	-	1.0	-
No	1.355	0.348	3.8 (1.9–7.7)	<0.001
**Child’s prophylaxis status**				
Yes	-	-	1.0	-
No	2.049	0.779	7.8 (1.7–35.7)	0.009

The analysis was limited to complete cases only (i.e., no missing data on five risk factors included in the final multivariable analysis of 2253 children (292 were HIV-infected).

## Discussion

The international community has responded to the launch of the Global Plan to eliminate HIV MTCT by 2015 [13]. More pregnant women have been and will be screened for HIV, and more HIV-exposed infants have been and will be tested for HIV infection. The pediatric risk of HIV infection could be reduced to less than 5% by 2015 through PMTCT interventions [14]. The goal of this study was to evaluate the Senegalese national PMTCT program after the increases in both the PMTCT services implemented in primary health care facilities and the accessibility of EID due to the use of DBS sampling from 2008 to 2015.

This report showed that Senegal has attained virtual eMTCT due to programmatic efforts related especially to greater access to EID testing, treatment of mothers and prophylaxis of infants.

### Global prevalence

The rate of MTCT decreased from 14.8% to 4.1% between 2008 and 2015, certainly due to a great intensification of efforts towards PMTCT services in Senegal via a combination of factors, including the increase in the number of PMTCT sites, which increased from 258 in 2008 to 1146 in 2015. In addition, the use and scaling up of DBS sampling for EID since 2007, task shifting to provide rapid screening tests in decentralized settings in 2007, task shifting to provide antiretroviral drugs (ARV) in decentralized settings in 2010, and the adoption of Option B+ while developing microplans for eMTCT in 2012 are likely the main contributors to this effort.

All these factors have contributed to the decrease in the rate of MTCT in Senegal, as in other countries [15–17]; the greatest progress in reducing new infections was seen in 2015 for Uganda, South Africa, Burundi, Namibia, Mozambique, Botswana, and Swaziland due to the rapid increase of integrated PMTCT services [18]. Despite this success in Senegal, a further reduction is needed to truly move towards the elimination of MTCT (< 2%), a goal already achieved by other countries. Universal access to integrated PMTCT services led to eMTCT in Cuba in mid-2015 and in Thailand, Belarus, Armenia and the Republic of Moldova in 2016 [19–21].

However, these programmatic data could be biased due to the lack of information regarding prophylaxis and feeding modes in 2008 and 2009. Those missing data, added during some years of the study, showed that additional effort needs to be made to improve data collection and management system. Moreover, we cannot exclude the possibility that EID testing was offered mainly to symptomatic children, which could explain the high rate of HIV infection observed during those years. Moreover, the declining MTCT rate between those years was due to the scaling up of PMTCT program illustrated in Fig 1 and the decreased number of sick children tested in PMTCT.

### HIV transmission-associated factors

As reported in other studies, factors associated with HIV transmission were a late age at diagnosis, the lack of ARV provision for both mother and child, and feeding with the exclusive/mixed modes. In this study, in addition to the year-to-year increase in the number of PMTCT sites, factors contributing to the reduction in MTCT were earlier testing and the increased treatment of mothers/prophylaxis of infants to protect against HIV infection from breastfeeding. Indeed, the incidence of earlier testing improved over the years; an increased number of children were tested before 3 months of age, and these children had an HIV infection rate 2 to 9 times lower than that estimated for children tested after 3 months of age. However, data regarding the time lag in returning the results to the patients were missing in this analysis, and the impact of this lag on the timely initiation of ART could not be assessed. Indeed, the delayed diagnosis of HIV-exposed infants due to the delayed receipt of the collected samples and the delayed return of the results to the testing site will in turn delay treatment initiation [22–26]. The results from several studies [22, 27–29] highlighted the importance of implementing efficient strategies, like the integration of innovative POC instruments into the conventional laboratory diagnostic network, to facilitate sample collection and timely results reporting [30]. However, EID has been found to be an excellent indicator for evaluating PMTCT success in some countries in SSA, as it was in this study [22, 31–32].

The proportion of breastfeeding mothers on HAART increased over time, and the impact of breastfeeding with ART on reducing MTCT was 10 times more significant than that of formula feeding, as has been described in other studies in African countries [33–36].

When considering treatment of mothers and/or prophylaxis of children, we found that the significant improvement in the provision of ARV to infected pregnant women and exposed children was associated with a decrease in the MTCT rate over the study period. However, the study confirmed that the lack of treatment and/or child prophylaxis remain the main factor correlated with vertical transmission, as has been shown in several countries where ARV intervention was late or unsatisfactorily implemented [29, 37–41], and highlighted the benefits of antenatal HAART (Option B+) in reducing the risk of MTCT [42].

However, in this study, no information was available regarding the adherence of the mothers to treatment or that of the children to prophylaxis, which is a broader challenge and limitation when measuring the effectiveness of PMTCT programs.

## Conclusion

This report indicated the effectiveness of PMTCT in Senegal, showing that the MTCT rate decreased to less than 5% between 2008 and 2015. This decrease could be due to the greater and earlier access to EID allowed by DBS sampling combined with an increase in PMTCT services. Task shifting to integrate primary health care centers, the adoption of Option B+ for pregnant women, the improved coverage of antiretroviral prophylaxis for babies, and the use of breastfeeding on ART were probably the key factors underlying the improved organization of maternal and infant health care services. However, due to the possible further postpartum infection of uninfected newborns via breastfeeding, efforts must be strengthened—especially towards improving the adherence of mothers to treatment and children to prophylaxis, enhancing counseling and monitoring infant feeding in order to achieve the goal of an MTCT rate of less than 2% by 2020 for a generation without AIDS in 2030.

## Supporting information

S1 File Data underlying this study. (XLS)
